# From little things big things grow: enhancement of an acoustic telemetry network to monitor broad-scale movements of marine species along Australia’s east coast

**DOI:** 10.1186/s40462-024-00468-8

**Published:** 2024-04-23

**Authors:** Adam Barnett, Fabrice R. A. Jaine, Stacy L. Bierwagen, Nicolas Lubitz, Kátya Abrantes, Michelle R. Heupel, Rob Harcourt, Charlie Huveneers, Ross G. Dwyer, Vinay Udyawer, Colin A. Simpfendorfer, Ingo B. Miller, Tracey Scott-Holland, Carley S. Kilpatrick, Samuel M Williams, Daniel Smith, Christine L. Dudgeon, Andrew S. Hoey, Richard Fitzpatrick, Felicity E. Osborne, Amy F. Smoothey, Paul A. Butcher, Marcus Sheaves, Eric E. Fisher, Mark Svaikauskas, Megan Ellis, Shiori Kanno, Benjamin J. Cresswell, Nicole Flint, Asia O. Armstrong, Kathy A. Townsend, Jonathan D. Mitchell, Matthew Campbell, Victor M. Peddemors, Johan A. Gustafson, Leanne M. Currey-Randall

**Affiliations:** 1 Marine Data Technology Hub, James Cook University, Townsville, QLD 4811 Australia; 2 Biopixel Oceans Foundation, Cairns, QLD 4878 Australia; 3 Integrated Marine Observing System (IMOS) Animal Tracking Facility, Sydney Institute of Marine Science, Mosman, NSW 2088 Australia; 4 School of Natural Sciences, Macquarie University, North Ryde, Sydney, NSW 2109 Australia; 5 Australian Institute of Marine Science, Townsville, QLD 4810 Australia; 6 Integrated Marine Observing System, University of Tasmania, Hobart, Tas 7001 Australia; 7 College of Science and Engineering, Flinders University, Adelaide, SA 5042 Australia; 8 School of Science, Technology and Engineering, University of the Sunshine Coast, Sunshine Coast, QLD 4556 Australia; 9 Australian Institute of Marine Science, Darwin, NT 0810 Australia; 10 University of Tasmania, Hobart, Tas 7001 Australia; 11 College of Science and Engineering, James Cook University, Townsville, QLD 4811 Australia; 12 Queensland Department of Agriculture and Fisheries, Brisbane, QLD 4000 Australia; 13 Queensland Government, Department of Environment and Science, Queensland Parks and Wildlife Service, Manly, QLD 4000 Australia; 14 Department of Primary Industries, Fisheries Research, Sydney Institute of Marine Science, New South Wales, Mosman, NSW 2088 Australia; 15 Department of Primary Industries, New South Wales, National Marine Science Center, Southern Cross University, Coffs Harbour, NSW 2450 Australia; 16 GBR Biology, Experience Co., Cairns, QLD 4870 Australia; 17 Dalrymple Bay Coal Terminal, Haypoint, Mackay, QLD 4740 Australia; 18 Gladstone Ports Corporation Limited, Gladstone, QLD 4680 Australia; 19 Coastal Marine Ecosystems Research Centre, Central Queensland University, Rockhampton, QLD 4702 Australia; 20 Coastal and Marine Research Centre, Griffith University, Gold Coast, QLD 4215 Australia; 21 AIMS@JCU, Division of Research and Innovation, James Cook University, Townsville, QLD 4811 Australia

**Keywords:** Animal movement, Spatial ecology, Drivers of migration, Migratory patterns, Residency, Sharks, Fish

## Abstract

**Background:**

Acoustic telemetry has become a fundamental tool to monitor the movement of aquatic species. Advances in technology, in particular the development of batteries with lives of > 10 years, have increased our ability to track the long-term movement patterns of many species. However, logistics and financial constraints often dictate the locations and deployment duration of acoustic receivers. Consequently, there is often a compromise between optimal array design and affordability. Such constraints can hinder the ability to track marine animals over large spatial and temporal scales. Continental-scale receiver networks have increased the ability to study large-scale movements, but significant gaps in coverage often remain.

**Methods:**

Since 2007, the Integrated Marine Observing System’s Animal Tracking Facility (IMOS ATF) has maintained permanent receiver installations on the eastern Australian seaboard. In this study, we present the recent enhancement of the IMOS ATF acoustic tracking infrastructure in Queensland to collect data on large-scale movements of marine species in the northeast extent of the national array. Securing a relatively small initial investment for expanding receiver deployment and tagging activities in Queensland served as a catalyst, bringing together a diverse group of stakeholders (research institutes, universities, government departments, port corporations, industries, Indigenous ranger groups and tourism operators) to create an extensive collaborative network that could sustain the extended receiver coverage into the future. To fill gaps between existing installations and maximise the monitoring footprint, the new initiative has an atypical design, deploying many single receivers spread across 2,100 km of Queensland waters.

**Results:**

The approach revealed previously unknown broad-scale movements for some species and highlights that clusters of receivers are not always required to enhance data collection. However, array designs using predominantly single receiver deployments are more vulnerable to data gaps when receivers are lost or fail, and therefore “redundancy” is a critical consideration when designing this type of array.

**Conclusion:**

Initial results suggest that our array enhancement, if sustained over many years, will uncover a range of previously unknown movements that will assist in addressing ecological, fisheries, and conservation questions for multiple species.

**Supplementary Information:**

The online version contains supplementary material available at 10.1186/s40462-024-00468-8.

## Background

Connectivity, through the movement of animals and/or dispersal of larvae, is a fundamental ecological and evolutionary process [1–3]. Connectivity not only influences the population trajectory of a species over space and time [1, 4], it has become increasingly important for preserving biodiversity and migratory movements [5, 6]. Understanding seasonal and ontogenetic patterns of movement and connectivity can help identify habitats essential for supporting specific functions such as reproduction, feeding and/or growth throughout a species’ life history [7–9]. Furthermore, determining the degree of connectivity is central to ascertaining whether a particular species within a given geographic region consists of a single widespread population or multiple discrete stocks [4, 10]. Information on connectivity and habitat use are also important for understanding resilience of populations to anthropogenic impacts such as climate change and re-stocking ability after overfishing, as well as managing human-wildlife conflict. As episodic events driven by global warming increase in frequency and severity [11], there has been a significant shift of highly mobile marine species’ biodiversity away from the equator [12–14]. This highlights the need to better understand the drivers of migration and essential habitat use, and to monitor the movements of species over large spatial scales and long time periods.

Over the last two decades, acoustic telemetry has proven an effective tool to monitor the movement and distribution of aquatic species due to its relative low cost, ease of use, reliability, ability to track individual animals over long periods (e.g., > 10 years), and compatibility across studies [15]. Acoustic receiver array designs vary from grid patterns aimed at maximising coverage of specific areas [e.g. within marine reserve boundaries; 16], to arrangements that encircle geomorphological features such as offshore reefs or seamounts [17, 18], and large-scale arrays that incorporate receiver gates or curtains to record movements among embayments, estuaries [19], or along coastlines [e.g. 20, 21, 22]. Large-scale networks of acoustic receivers are increasingly expanding across the world’s oceans, forming regional- to continental-scale arrays that can address a broader range of ecological questions, including the identification of long-range movements or population connectivity [10, 23, 24]. These networks can vary from groups of collaborating local researcher-led arrays that share data [e.g. 25, 26], to large-scale coordinated networks that combine research-led and backbone infrastructure (i.e. the ongoing-permanent deployment of a receiver or group of receivers at a specific locality) and a central database to facilitate data sharing. Australia’s Integrated Marine Observing System’s Animal Tracking Facility (IMOS ATF) [27, 28], Canada’s Ocean Tracking Network [23], the Florida Atlantic Coast Telemetry Network, and South Africa’s Acoustic Tracking Array Platform [24, 29] are examples of such networks. In those networks, array configuration is a complex balance of operational design, logistics, and implementation costs [30], where multiple institutes and collaborators are almost inevitably required to cost-effectively maintain large arrays.

Since 2007, IMOS ATF has maintained a set of strategically located, permanent, backbone receiver installations around Australia to detect broad-scale and cross-jurisdictional movements of marine species [28]. Additional site-specific installations are operated by individual research groups and contributed to enhance the collaborative network [27]. The configuration and longevity of these researcher-led installations vary depending on the research needs of each group. Given that independent research projects often have defined commencement and completion dates, and vary in deployment duration, the receiver coverage in Australia has changed over the years [28]. So far > 12,400 receivers have been deployed at > 240 locations nation-wide (animaltracking.aodn.org.au). At the time of writing, the IMOS collaborative telemetry network comprised over 1,200 receivers around Australia, about one third of which is maintained permanently by the IMOS ATF, with the remainder operated by individual research groups. On the east Australian seaboard alone, the IMOS ATF network is made up of 462 receivers deployed across 3,000 km of coastline and three state jurisdictions from Tasmania (42.7°S) to northern Queensland (11.4°S).

In this study, we present a recent enhancement of the IMOS east coast acoustic tracking infrastructure in Queensland waters, northeast Australia (the northern 2,100 km of the IMOS network), and its benefits in improving the capacity to collect data on broad-scale movements of marine species. Specifically, we compare the benefits of the new infrastructure by contrasting the acoustic receiver array originally in place by the IMOS ATF and other groups (referred to as *existing array*) with the additional array deployed from 2019 by a Queensland project (referred to as *new array*). We analysed data collected between 2019 and 2022 to (1) describe the design of the *new array* and how it enhanced receiver coverage on the east coast of Australia; (2) quantify the increase in coverage and the additional detections provided by the additional receivers; and (3) highlight examples of new information gained regarding the movement ecology of representative elasmobranch and teleost species. Finally, (4) we highlight the collaborative momentum generated by this initiative and discuss its limitations, benefits, and the lessons learnt. These insights are relevant to other large-scale acoustic tracking efforts and to future studies interested in using this approach.

**Table:** 

Box 1. Terminology	
Existing array	Acoustic receivers deployed in Queensland by IMOS Animal Tracking Facility and collaborating research groups
New array	Acoustic receivers deployed as part of the ‘Queensland IMOS Acoustic Telemetry Array Project’ 2019–2022
Enhanced Queensland array	All receivers deployed in Queensland waters, encompassing the existing and new arrays
National array	Australia-wide network of acoustic telemetry infrastructure (permanent receivers and data base)

## Enhancing acoustic receiver coverage in Queensland waters: a new array design

Despite extensive receiver coverage and the large number of animals tagged on the east coast of Australia, the IMOS ATF backbone network configuration includes gaps. Permanent IMOS ATF infrastructure in Queensland historically included a small number of receiver curtains or arrays around specific islands on the Great Barrier Reef (e.g., Orpheus Island, Heron Island), primarily due to the logistical challenges of maintaining equipment across this vast region in a sustained manner. The resultant receiver configuration left large spatial gaps in coverage along the Queensland coastline and across the many offshore reef islands, thereby limiting the ability to track the movements and distributions of mobile or migratory species in detail.

In 2019, additional funding from the Queensland Government enabled the strategic expansion of the IMOS acoustic receiver infrastructure into coastal and offshore Queensland waters, to improve the collection of data on broad-scale movements of marine species. This included deployment of receivers at beaches monitored by the Queensland Shark Control Program and at Australian Institute of Marine Science (AIMS) monitoring sites, and was complemented by a tagging program targeting elasmobranch and teleost species of commercial value, conservation concern, and/or human interest (e.g., responsible for shark bites).

**Fig. 1:**
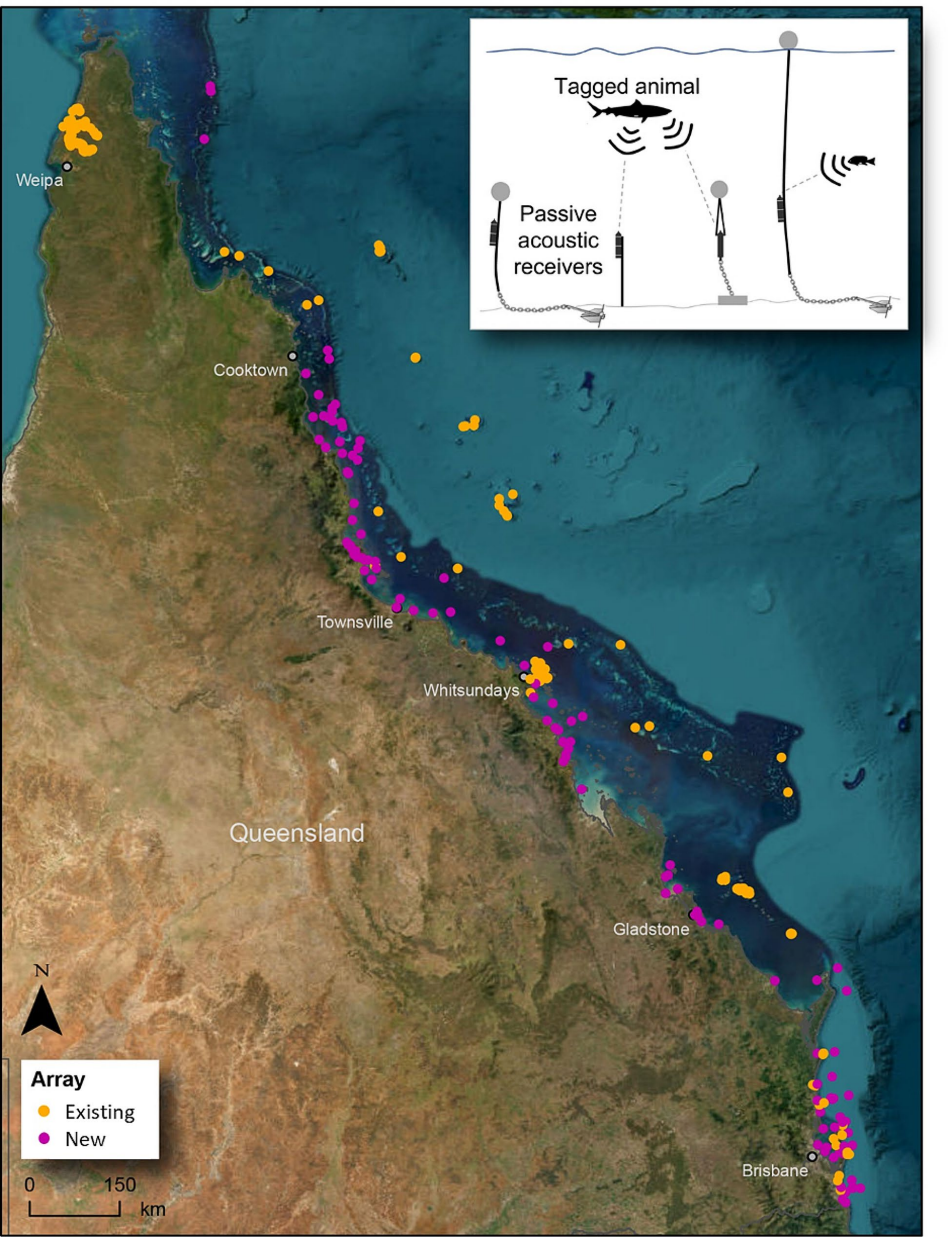
Map of Queensland, Australia, showing the locations of acoustic receiver deployments making up the existing (orange) and new (pink) arrays. The inset illustrates four of the five main installation methods i.e. subsurface floats with anchor/weights, star pickets, subsurface floats with anchor/weights with an acoustic release, and surface float setups with anchor/weights. Receivers are also attached directly to underwater infrastructure e.g., wharfs, sensor equipment

In Queensland, the existing array consisted mainly of local grids and curtains (referred to as the ‘*existing array*’), which included 208 acoustic receiver stations operated by IMOS ATF and collaborating research groups, in place between July 2019 and July 2022 (Table 1). The *existing array* includes installations such as the North Stradbroke Island receiver curtain, the Heron Island and One Tree Island arrays, and various local installations maintained by individual research groups, e.g., in rivers and estuaries in the Gulf of Carpentaria and the Sunshine Coast, around Lady Elliot Island, the Whitsundays, North West Island, and Coral Sea seamounts, at reefs along the Great Barrier Reef, and at grey nurse shark *Carcharias taurus* aggregation sites in southern Queensland (Fig. 1).

**Table 1:** Number of receiver stations and organisations managing the receivers for the *existing* and *new array*

Existing array collaborator	no. stations	New array collaborator	no. stations
UniSC/UQ/RT/Australia Zoo (Research/Industry/Tourism)	63	QDAF Fish Aggregation Devices (FADs) Program (Government)	18
JCU Fish and Fisheries (Research)	32	QDAF Queensland Shark Control Program (Government)	18
BOF/QDAF (Research/Government)	28	JCU Marine Data Hub (Research)	14
QPWS & P (Government)	24	QPWS & P (Government)	14
JCU/MBD (Research/Tourism)	19	MMP (Government/Research)	12
IMOS ATF (Research)	17	JCU/BOF/QDAF/DBCT (Research/Government/ Industry)	10
IMOS ATF/AIMS LTMP (Research)	15	AIMS (Research)	10
Griffith University (Research)	6	Experience Co. Cairns (Tourism)	9
IMOS ATF/Project Manta (Research)	4	BOF (Research)	7
		CQU/GPC (Research/Industry)	4
		Griffith University (Research)	1
		Yongala Dive (Tourism)	1
		Seagrass Ecology Group JCU (Research)	1
**Subtotal**	**208**		**119**
**Total Enhanced Qld Array**			**327**

AIMS = Australian Institute of Marine Science; BOF = Biopixel Oceans Foundation; CQU = Central Queensland University; DBCT = Dalrymple Bay Coal Terminal; DES = Department of Environment & Science; GPC = Gladstone Ports Corporation; IMOS ATF = Integrated Marine Observing System-Animal Tracking Facility; JCU = James Cook University; AIMS LTMP = AIMS Long Term Monitoring Program; MBD = Mike Ball Dive Expeditions; MMP = Marine Monitoring Program; QDAF = Queensland Department of Agriculture & Fisheries; QPWS & *P* = Queensland Parks and Wildlife Service; RT = Rio Tinto; UQ = The University of Queensland; UniSC = University of the Sunshine Coast.

The state-wide enhancement of acoustic receiver coverage led to 119 additional receivers (‘*new array*’) deployed from the Gold Coast (28.15° S, 153.53° E) on the state’s southern border with New South Wales to remote far north Queensland (11.42° S, 144.03° E), spanning 2,100 km, or 16.7° latitude (Fig. 1). Unlike much of the existing infrastructure, and to maximise the monitoring footprint whilst minimising logistical costs, this new initiative often included single receivers deployed at specific sites or stations, and maintained by a range of collaborators or co-investment partners operating in specific regions. These new receiver stations were positioned to fill gaps in coverage between existing installations, particularly in North Queensland. Fourteen agencies collaborated to deploy and maintain the *new array*, which is coordinated through the Australian Institute of Marine Science (AIMS), James Cook University (JCU), and IMOS ATF. Collaborating organisations include research institutes, universities, government departments, port corporations, port-side industries, and tourism operators (Table 1).

**Fig. 2:**
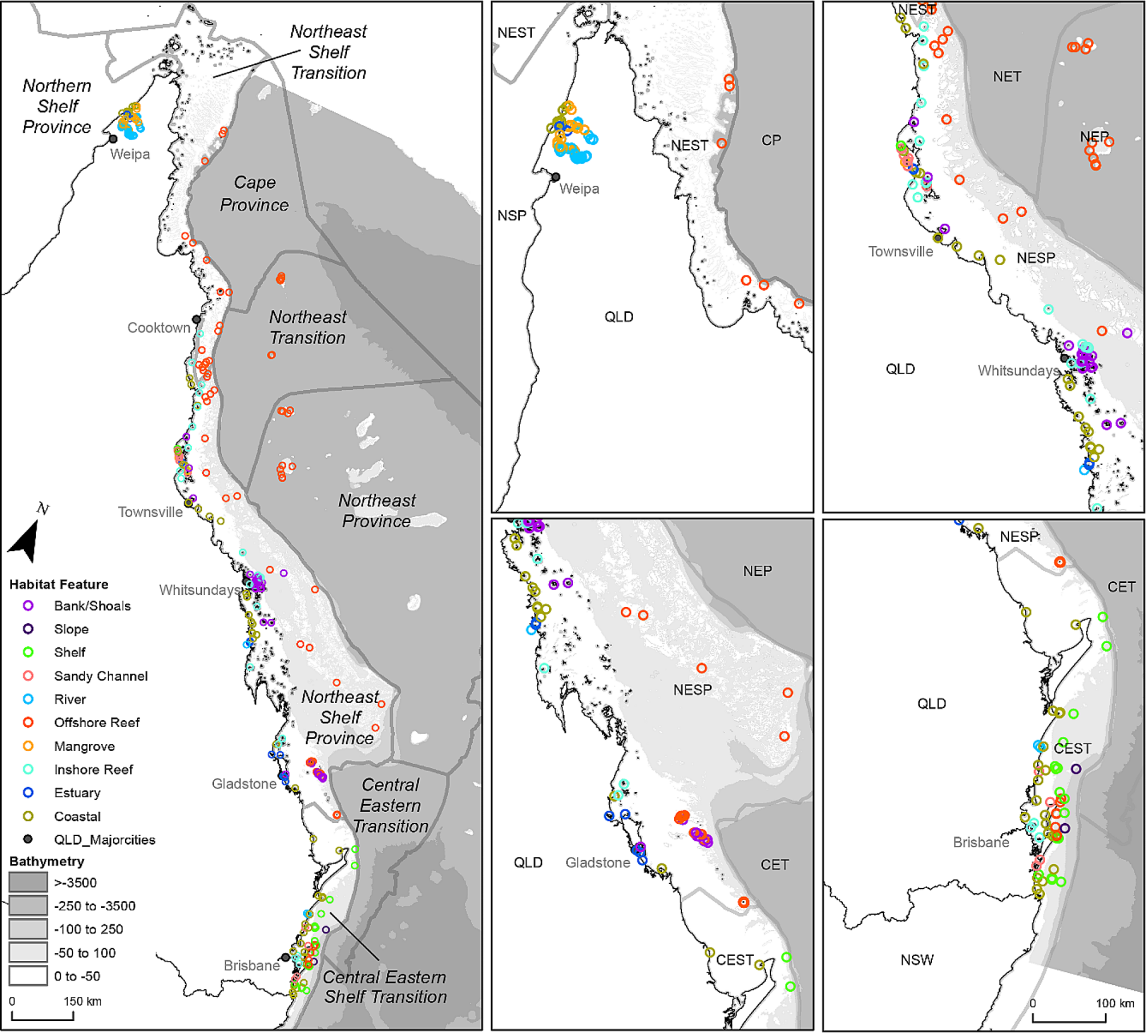
Receiver locations coloured according to the predominant, broad-scale habitat features, overlayed on Queensland’s provincial marine bioregions. Abbreviations: Northern Shelf Province (NSP), Northeast Shelf Transition (NEST), Cape Province (CP), Northeast Transition (NET), Northeast Province (NEP), Northeast Shelf Province (NESP), Central Eastern Transition (CET), Central Eastern Shelf Transition (CEST)

Together, the *existing array* and *new array* (hereafter collectively referred to as the *‘enhanced Queensland array’*) comprise 327 receiver stations deployed across a broad range of habitats and bioregions (Fig. 2). This enhanced infrastructure constitutes the most comprehensive acoustic receiver network and offers the broadest spatial coverage in Queensland waters to date. Combined with the receiver infrastructure already in place in the neighbouring states of New South Wales, Victoria, and Tasmania, the receiver coverage along the east Australian coast was increased to almost 4,000 km, substantially value-adding to the national array coordinated by the IMOS ATF. All data from both the *existing* and *new array* are made publicly available through the IMOS Animal Acoustic Tracking Database (animaltracking.aodn.org.au), providing valuable data and timely notifications to researchers when their tagged animals are detected by collaborating projects across the network.

## Effectiveness of the enhanced Queensland array

The benefits and effectiveness of the *enhanced Queensland array* were assessed by (1) comparing the acoustic receiver coverage across provincial bioregions (DCCEEW, Supp 1) and habitats between the existing and new arrays; (2) comparing the overall number of detections and number of animals detected between the *existing* and *new arrays*; and (3), for representative species, examining how detectability and movement metrics changed as a result of the enhanced receiver coverage. Movement and detectability metrics for this subset of species were calculated (i) using only receivers in the *existing array* configuration, and (ii) using all receivers in the *enhanced Queensland array* (i.e. *existing* plus *new arrays*). The analysis used the same individuals, which were all tagged after the deployment of the new array (post July 2019), with metrics calculated including the (a) proportion of tagged individuals detected on both array configurations, (b) number of days detected for each individual, (c) maximum distance travelled between any two detections, and (d) latitudinal range of movements captured by both array configurations. Mean values were then calculated for each of these metrics and plotted to assess differences in species-level movement metrics estimated using the two array configurations.

### Receiver coverage

**Fig. 3:**
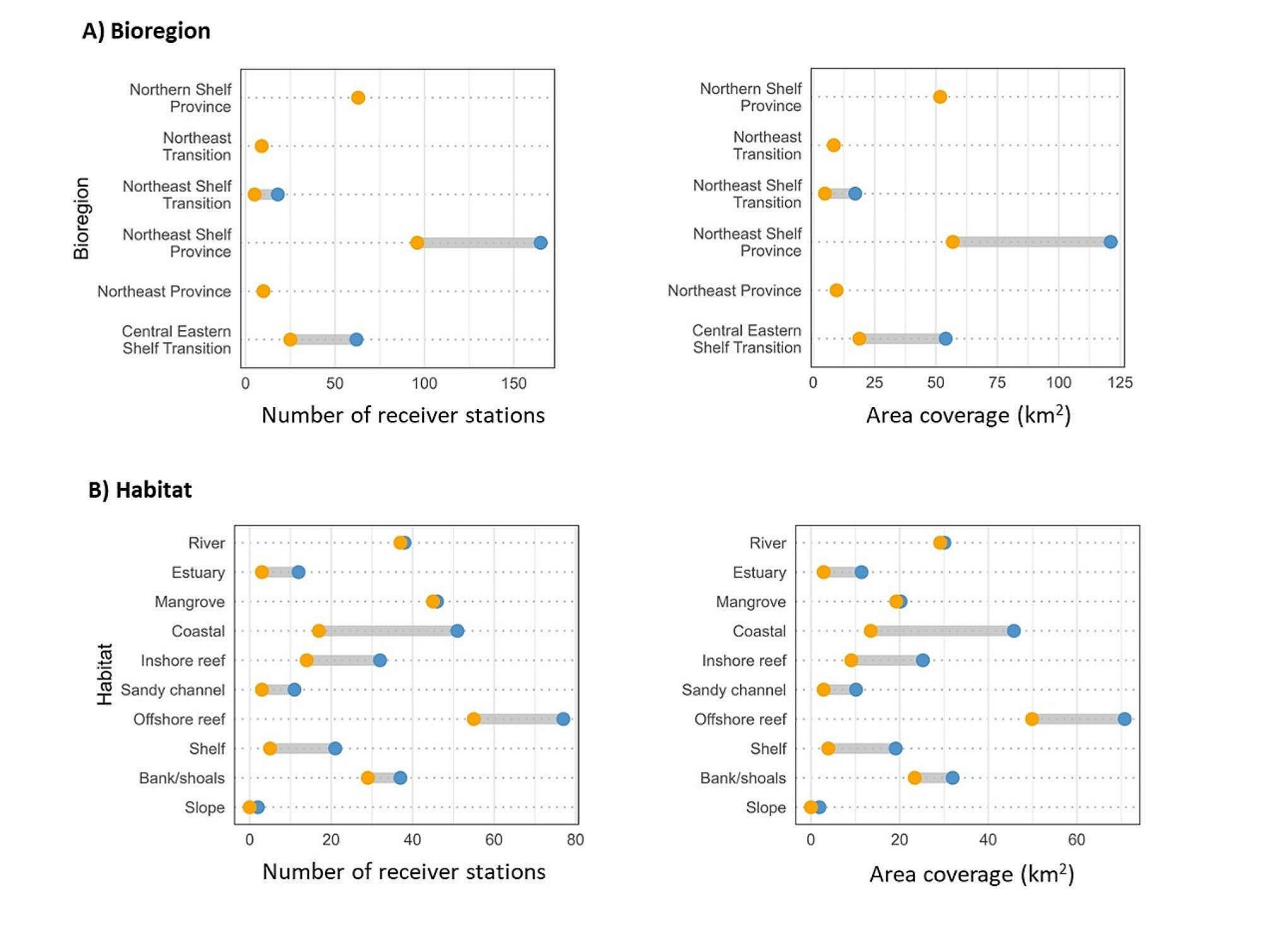
Increase in number of receiver stations (left panels) and receiver coverage (right panels) between the *existing array* (orange) and the *enhanced Queensland array* (blue), for the different provincial marine bioregions (**A**) and habitats (**B**)

The *new array* increased the total number of acoustic receivers deployed in Queensland waters from 208 to 327, representing a 57% increase. Deployment effort for the new array was primarily concentrated in coastal areas and driven by opportunity and logistics. As a result, no additional receivers were deployed in the Northern Shelf, Northeast Transition, and Northeast Province bioregions (Figs. 2 and 3A). This was due to those areas being difficult to access and maintain receiver moorings, as they transition in depths from the epipelagic to the bathypelagic zone (100–2,000 m). With the *new array*, the number of receivers in the Northeast Shelf Province nearly doubled, increasing from 96 to 165 (Fig. 3A). There was also an increase in number of receivers in the Central Eastern Shelf Transition (25 to 62) and in the Northeast Shelf Transition (5 to 18) provincial bioregions. The *new array* also covered some habitats more than others. There were limited additional receivers in river (*n* = 1), mangrove (*n* = 1), and slope (*n* = 2) habitats, while the number of receivers in estuary, sandy channel, shelf, and offshore reef habitats more than doubled, and nearly tripled for coastal habitat (Fig. 3B). As there was no consistent source of fine-scale habitat data for the entire array, available spatial layers and personal observations of habitat were compiled to interpret broad-scale representation (Table S1).

Using a 550 m buffer as a proxy for the estimated detection range of each station [31], total receiver coverage increased by 75% (from 151 km^2^ to 265 km^2^). Receiver coverage was calculated in ArcMap (v10.8.1, *ESRI*) using the buffer tool and considering receiver overlap in detection range by dissolving any overlap. While receivers were clustered closer together in some locations and more spread out in others, patterns in number of receiver stations among bioregion and habitat categories were similar to patterns in receiver coverage (Fig. 3). The only notable difference was for mangrove environments, for which there were more receivers but lower spatial coverage compared to banks/shoals habitat (Fig. 3).

### Animal detectability

**Fig. 4:**
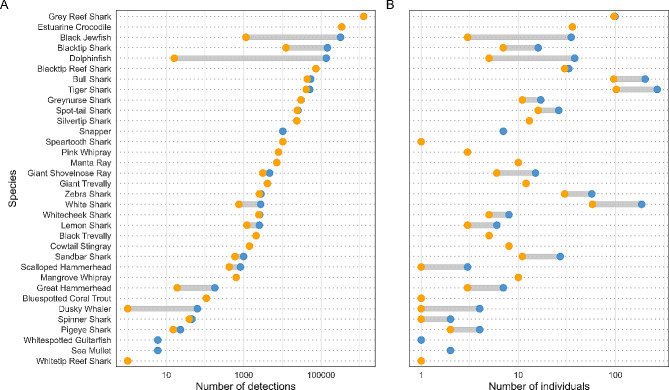
Number of detections (**A**) and number of individuals (**B**) recorded by existing array (orange) and the enhanced Queensland array (blue) for each species

Detection data recorded between July 2019 and June 2022 were downloaded from the Australian Animal Acoustic Telemetry Database (https://animaltracking.aodn.org.au/) and processed in the R statistical environment [32]. The R package REMORA [33] was used to filter out erroneous detections, assisting with quality assurance/quality control. A total of 2,531,148 detections across 222 acoustic receiver stations were recorded by the *Queensland array*. Species detected represent individuals tagged by 18 research projects based in Queensland, New South Wales, and South Australia (Table 2). The new array comprised 25% of the total detections recorded (624,028), yet of the 913 animals detected, 63% (576) were detected on new receivers, representing 68% (23 of the 34) of the total species detected. The new receiver stations substantially contributed to overall detections of several species, in particular, capturing almost 100% of the black jewfish *Protonibea diacanthus* (316,428 of 317,574 detections) and dolphinfish *Mahi mahi* (136,704 of 136,720 detections) detections (Fig. 4A). This is likely because these species were primarily tagged close to *new array* receivers. Similarly, grey reef sharks *Carcharhinus amblyrhynchos* and estuarine crocodiles *Crocodylus porosus* tagged in the remote Coral Sea and eastern Gulf of Carpentaria, respectively, were almost 100% detected by the *existing array* (Fig. 4), likely due to their predominantly resident behaviours in the areas they were tagged, coupled with the relative isolation of those receiver clusters [18, 34] (Fig. 4).

**Table 2:** Number of species tagged by 19 collaborating research projects that were detected across the enhanced Queensland array

Project	No. species detected
IMOS/AIMS Acoustic Telemetry Array Queensland	23
QDAF Whitsundays: Prevalence and behaviour of sharks	9
Orpheus Island Mangrove Array	6
NSW DPI (Whaler, White and Tiger Shark Program)	5
Eastern Gulf of Carpentaria Array	3
Queensland Shark Control Program	2
QPWS & P - Grey Nurse Shark-Diver Interactions	1
Leopard shark ecology, informing re-stocking program in Indonesia	1
IMOS-ATF Heron Island	1
JCU - North Queensland tiger shark tracking	1
JCU - Coral Sea	1
NSW DPI - SEACAMS	1
NSW DPI - Movements of Coastal Sharks	1
NSW DPI - Shark Meshing Program	1
Norfolk Island project	1
Southeast Queensland shark movement	1
Tracking of *Mahi mahi* in southeast Queensland	1
UniSC - Project Manta East Australia	1
White shark cage diving industry monitoring (South Australia)	1

The *new array* provided additional movement information for several other wide-ranging species, such as blacktip sharks *Carcharhinus limbatus/tilstoni*, bull shark *Carcharhinus leucas*, white shark *Carcharodon carcharias*, and tiger shark *Galeocerdo cuvier* (Fig. 4B). For some species, the new infrastructure substantially increased the number of detections (e.g., blacktip sharks, Fig. 4A) and the number of individuals detected (e.g., bull, tiger and white sharks; Fig. 4B).

#### Movement metrics

Comparisons in species-level movement metrics between the *existing* and *new array* are presented for six representative species. These include (1) species with different management importance, and (2) species tagged in both the new array and through independent projects. Four of those species (bull shark, tiger shark, giant shovelnose ray *Glaucostegus typus*, black jewfish) were tagged as part of the *new array* project. Bull and tiger sharks are responsible for a large proportion of shark bites in Australia [35, 36]. Giant shovelnose ray is a species of high global conservation concern for which Australia provides one of the last remaining strongholds [37]. Black jewfish, a commercially important teleost species, was targeted as a research priority to fill knowledge gaps in movement patterns, stock structure and post-release survival, after a rapid increase in commercial catch [38]. The other two species were the Indo-Pacific leopard shark *Stegostoma tigrinum* and the grey nurse shark *Carcharias taurus*, which were tagged by other agencies (University of Sunshine Coast, Queensland Parks and Wildlife Service & Partnerships, Sea World, and Biopixel Oceans Foundation). Both species are of high tourism value and conservation concern in Australia (grey nurse shark) and internationally (Indo-Pacific leopard shark) [16, 39–41].

**Fig. 5:**
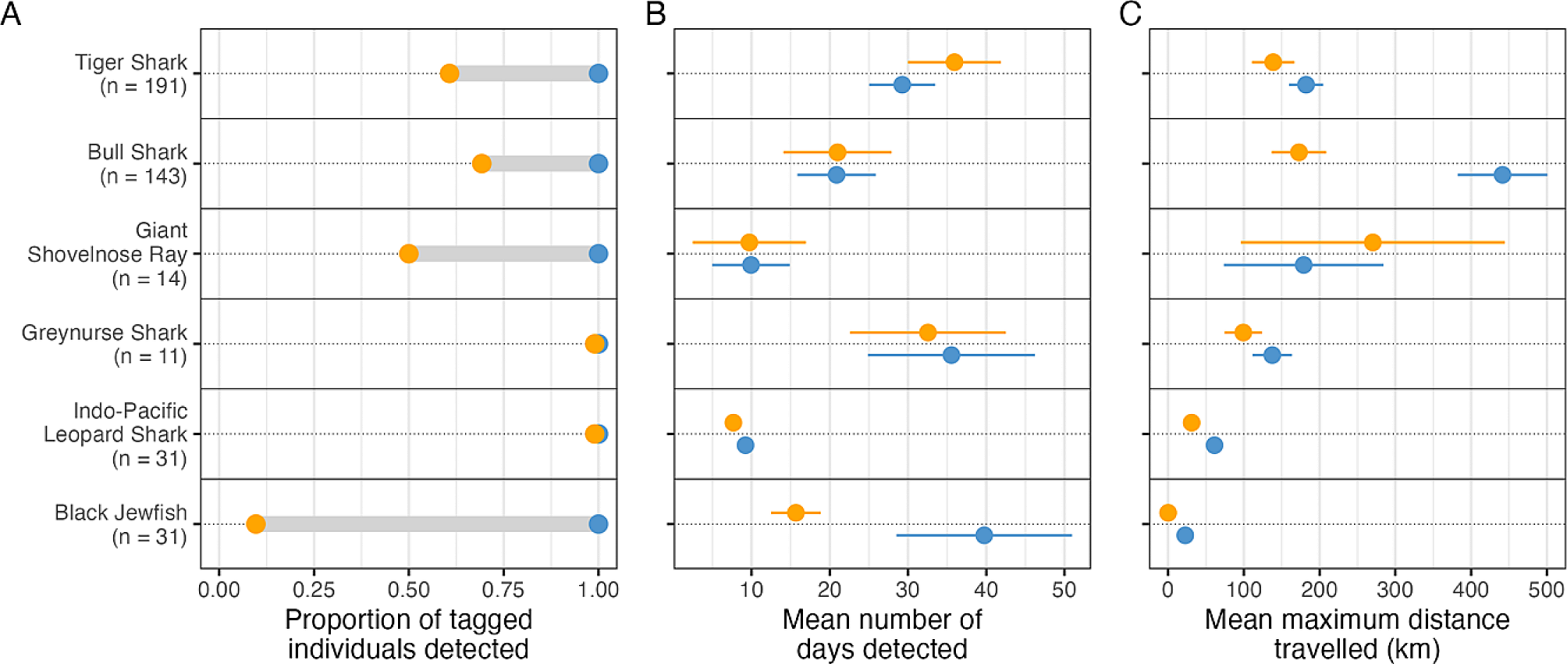
Summary movement metrics for a subset of six species tracked during the period of this study, comparing results based on the *existing* array (orange) with results based on the *enhanced Queensland array* (blue). Species are ordered from site attached (bottom) to highly mobile and migratory (top). Species-level mean (points) and standard error (whiskers) values are shown in panels B and C

Movement and detectability metrics were calculated for this subset of species i) using only receivers from the *existing* array configuration, and ii) using all receivers from the *enhanced Queensland array* (i.e., *existing* plus *new array*). The analyses used the same individuals, and metrics calculated included a) the proportion of tagged individuals detected on each array configuration, b) the number of days detected, and c) the maximum distance travelled between any two detection locations. The maximum distances travelled were estimated based on least-cost paths between consecutive detections. Least-cost paths were calculated using the ‘gdistance’ [42] and ‘terra’ [43] R packages, where a route between consecutive detection locations is calculated while considering any islands or coastlines occurring between the two locations. A spatial ‘cost’ grid (i.e., transition layer) was first computed using a high-resolution coastal shapefile (Geosciences Australia) where landmasses and islands were assigned a high ‘cost’ value (1000), and the ocean assigned low ‘cost’ value (0). The `shortestPath()` function was then used to calculate the shortest distance between consecutive detections by minimising the ‘cost’ along the path, with the algorithm allowing to transition between grid cells across all 16 possible directions to calculate the most realistic paths. These least-cost paths represent the shortest paths individuals could have moved through, thus providing the most conservative estimate of the distance individuals may have covered to fit the detection dataset. Mean values were then calculated for each metric, and plotted for comparison (Fig. 5).

Results from the *existing array* were used as a baseline to understand how the additional receivers provided by the *new array* adds to our understanding of movement for the six representative species. The changes in our estimation of species-level movement metrics between the existing and the *enhanced Queensland array* varied across species. For example, the proportion of tagged individuals detected increased for four of the six species (Fig. 5A), with negligible changes for grey nurse sharks and Indo-Pacific leopard sharks. The proportion of tiger shark, bull shark, and giant shovelnose ray detected increased by 25–50% with the addition of detections collected by the *new array*, while the proportion of black jewfish detected increased by more than 80%. With the increased detectability of individuals using the *enhanced Queensland array*, the other species-level movement metrics were refined, as more individuals were included in calculations. The mean maximum distance travelled changed the most for bull sharks, increasing by 2- to 3-fold in the *enhanced Queensland array*. This variation in the benefits of the *new array* across species shows the complexity of recording movement patterns of ecologically diverse species, but also highlights the different ways the *new array* can enhance our ability to understand the movements of marine species. For example, despite that bull sharks were not tagged in the far northern GBR, the addition of the most northerly receivers as part of the *new array* revealed larger migrations than previously thought (Fig. 5C).

For tiger sharks, the increase in number of individuals detected using the enhanced array was accompanied by a reduction in the mean number of days detected (Fig. 5A, B), as the increased detectability led to the inclusion of individuals that were more mobile and, therefore, detected on receivers less frequently. For giant shovelnose rays, the proportion of individuals detected substantially increased, while the mean maximum distance travelled marginally decreased (Fig. 5A, B), showing that the *new array* filled in gaps in spatial coverage leading to the detection of more individuals, while the longest distance movements were captured by the *existing array*. This highlights the combined benefits of both arrays, i.e., of the *enhanced array*. For grey nurse and Indo-Pacific leopard sharks, there was no change in number of individuals detected (Fig. 5A), but data captured by the *new array* further refined metrics of maximum distance travelled (5C), capturing long distance movements that could not have been detected with the *existing array* (Fig. 5C).

### Examples of large-scale movements detected in the Queensland array

The new array revealed previously unknown movements for several species along the east coast of Australia. Here, we present examples of previously unknown broad-scale movements detected by the *enhanced array* configuration for two sharks, a ray, and a teleost species. Results highlight the large dispersal capacities captured for each species using the *enhanced Queensland array*:

#### Bull shark

**Fig. 6:**
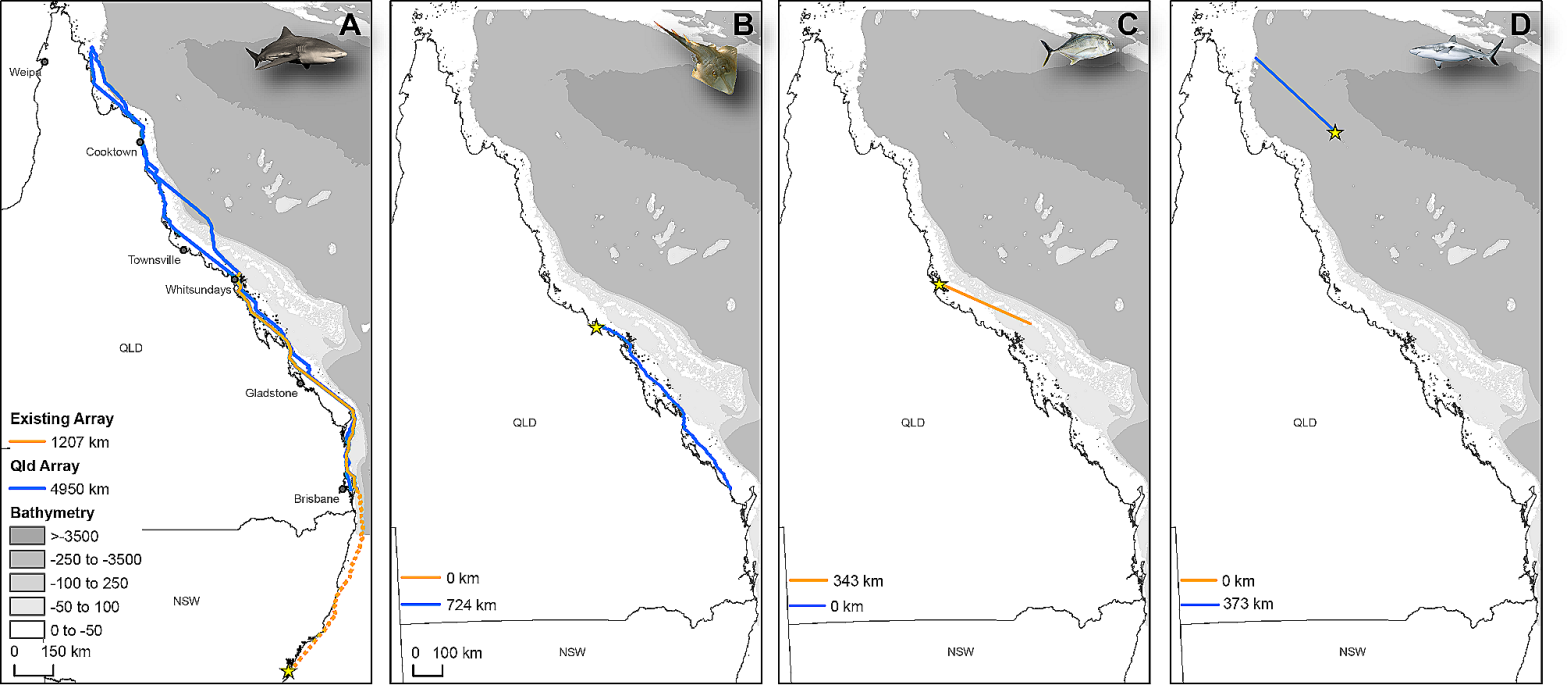
Individual movement trajectories captured from the arrays across the east coast of Australia. Distance travelled compares movements *in Queensland (QLD)* of each individual from only *existing* receivers (orange) and distance *combined existing and new* receivers (blue). Dotted orange line in panel A represents a single additional ∼ 2000 km movement from NSW to first receiver detected in the existing QLD array. For panels B and D, no movements were detected with only the *existing* array. Yellow stars represent tagging location. Panels: (**A**) bull shark, male total length (TL) 249 cm (total distance for existing array includes distance travelled from tag location in NSW to first receiver detected in QLD), (**B**) giant shovelnose ray, female TL 249 cm, (**C**) giant trevally, TL 57 cm, (**D**) grey reef shark, female TL 164 cm. Movements shown are based on least-cost paths between receivers

Previous acoustic tracking studies on the east coast of Australia revealed straight line dispersal distances of adult bull sharks of up to 1,770 km, between Sydney Harbour, New South Wales, and reefs off Townsville, Queensland [20]. The enhancement of the *existing array* into Far North Queensland extended this distance to at least ∼ 2,900 km, by detecting the movement of a bull shark tagged in New South Wales in Saunders Reef, in the northern limit of the *new array* (Fig. 6A).

#### Giant shovelnose ray

The spatial ecology of giant shovelnose rays is poorly understood. A previous study showed that adult giant shovelnose rays tagged in Cleveland Bay, Townsville, exhibited philopatric behaviour, leaving the bay for ∼ 9–12 months to an unknown destination [44]. The *enhanced Queensland array* detected an adult female giant shovelnose ray off Bundaberg, ∼ 725 km south of its initial tagging location in Abbot Bay (∼ 130 km southeast of Townsville; Fig. 6B). This represents the longest movement recorded for this species globally, and the first information on movements of giant shovelnose rays when absent from tagging locations on the east coast of Australia.

**Giant trevally***Caranx ignobilis*: Previous studies on giant trevally have focused on fine-scale movements and showed high site fidelity [45–48]. The *enhanced Queensland array* recorded a ∼ 350 km movement between Hinchinbrook Channel and the Whitsundays (Fig. 6C), which represents the longest movement recorded for this species in Australia. This adds to previous information of large-scale movements from Southern Africa of up to 633 km [48].

#### Grey reef sharks

Showing high residency to or near tagging locations, grey reef sharks are typically considered to be site attached, with limited examples of large-scale movements [18, 49, 50]. The largest movement previously reported was that of one sub-adult male, that undertook a ∼ 250 km round-trip between Osprey Reef in the Coral Sea and the Ribbon Reefs on the Great Barrier Reef [18, 51]. The *new array* detected another round-trip, from a mature female that moved between Osprey Reef and Saunders Reef in the northern Great Barrier Reef (Fig. 6D), further suggesting connectivity between the Coral Sea seamounts and the Great Barrier Reef, and that such movements might be more frequent than originally assumed. Completed within 25 days, this round-trip movement added up to ∼ 760 km, and is the longest movement recorded globally for the species.

## Accomplishments, lessons learnt, and future of the Queensland array

### The enhanced Queensland array initiative drives collaborative momentum

A highlight of the *enhanced Queensland array* is the collaborative momentum that has been built over the three years of this initiative. Four key benefits resulted from working collaboratively to develop this broad-scale array: (1) enhanced longevity of receiver deployments; (2) improved cost-effectiveness; (3) improved understanding of broad-scale movements of species tagged within, but also outside of, Queensland; and (4) provided learnings relevant to Queensland and other regions.

#### Enhanced longevity of receiver deployments

Large-scale arrays and associated research questions can suffer from the short-term nature of project-driven receiver deployments (i.e. receivers are removed at the end of studies with a finite funding period). Sites with regular access by collaborators initially helped to fill spatial detection gaps for the *new array*. Relationships established with collaborators with permanent access to certain sites allow receivers to be regularly maintained, so that the skeleton of the new array has the potential to remain deployed indefinitely. Given the number of collaborators involved in this project, it was important to ensure that the best practices in receiver handling were well known and consistently used. Furthermore, receivers were sent to collaborators ready for deployment (i.e. with new batteries and already programmed and initialised) and, when swapped, the old receivers were returned to the central management team for downloading and battery changes. Receiver set-up and downloading by the management team allowed for data quality control, ensuring successful and timely uploads into the IMOS database. Importantly, the receivers’ health could also be monitored and tested before redeployment.

#### Cost-effectiveness

Working with collaborators reduced the costs associated with servicing receivers, since receiver maintenance was performed during planned in-water activities, thereby reducing vessel fees and field work salaries. Multiple collaborators also enabled receivers to be placed at sites that would not be sustainable to manage for one project team. For example, three teams that quarterly–annually monitor their sites along the length of the Great Barrier Reef contributed to swapping over receivers, other receivers were serviced quarterly by a team that maintains vessel moorings at sites along the Queensland coast and by dive tourism operators at dive sites visited weekly. Maintenance, access, and funding for such an extensive array would have been impossible without the involvement of multiple teams and integration of this work into existing activities.

Another opportunity provided by the array and the developed collaborations was training. Exposure to the acoustic tracking technologies and use of equipment by staff within collaborator organisations can lead to additional research questions and projects, that can take advantage of the array infrastructure. For example, following the initial deployment of 10 receivers on fish aggregating devices (FADs) in southeast Queensland in collaboration with the Queensland Department of Agriculture & Fisheries (QDAF) primarily to study dolphinfish, 22 receivers are now maintained by the QDAF FADs Program, including at sites in the eastern Gulf of Carpentaria. Other examples of capacity building include the support of postgraduate projects (e.g., dolphinfish and shark species including great hammerheads [52, 53]) and a new study tracking sailfish involving collaborations with game fishing clubs. Such training can build a skilled workforce for the future, alleviating the reliance on a small number of people, and produce greater scientific and social impact. Moreover, the *enhanced Queensland array* and, indeed the national array coordinated by the IMOS ATF, has provided both infrastructure and data that students can use, but particularly contribute via provision of long-term datasets that cannot be collected in the timeframe of a typical undergraduate or postgraduate research project. The enhancement of the state-wide array and established collaborations provide great benefit to students and researchers seeking to start new studies in Queensland.

#### Improved understanding of species movements - conservation and management implications

The acquisition of new data provided by the *enhanced Queensland array* has benefited science and management outcomes. So far, tagged animals from 18 projects on the east coast of Australia have been detected on Queensland’s receivers. Local studies in Queensland have been complemented with large-scale and longer-term movement data when their tagged animals moved away from local arrays. Examples include a study aiming to understand shark residency and behaviour in relation to human activities in the Whitsunday Islands, which would normally only provide information in a local context. The *enhanced Queensland array* provided further data on when and where those species go, showing that some species move far beyond the locations where individuals were originally tagged [53]. A study into the conservation effectiveness of MPAs for protecting the critically endangered east Australian population of grey nurse sharks benefited from the additional detections away from focal aggregation sites, providing new information on where individuals go when they disperse away from protected area boundaries and could aid in finding new aggregation sites [16]. In another example, information on movement ecology and post-release survival gained from detections of black jewfish contributed to a recent fisheries stock assessment [41]. Likewise, tiger sharks, bull sharks, and white sharks tagged by colleagues in the neighbouring state of New South Wales were regularly detected throughout the *enhanced Queensland array* (Table 2), including white and tiger sharks detected at Coral Sea sites, providing greater insights into the movement patterns of these highly mobile species of high human interest.

#### Lessons learnt, compromises, and considerations

The *enhanced Queensland array* has an atypical design compared to other large-scale movement studies. There are minimal acoustic curtains in this array, and installations of multiple receivers are limited to a few locations. Instead, the array has many single receivers spread out across the State and out into the Coral Sea. Given the design of the array, there are compromises to consider (Table 3).

**Table 3:** Compromises and advantages of the Queensland single-array design

Compromises	Advantages
Receiver deployments can be limited to the sites and depths where collaborators operate (e.g., sheltered sections at the back of reefs and islands; 5–9 m depth), which might not be optimal for detection range and might not be the best location where animals are most likely to swim past.	Receivers have the potential to be maintained indefinitely by collaborators, so the skeleton of the large-scale array remains in place, thereby reducing the impact that changes in receiver placement can have on large-scale movement studies.
Animals may not be tagged near a receiver, or in a region with only one receiver. Risk of collecting very little data for some species if not enough individuals move to a region with more receivers or swims past single receivers.	Ability to tag at many more locations within the *enhanced Queensland array* skeleton (e.g., multiple locations to address connectivity and stock structure questions).
Single receiver deployments may not detect the same number of individuals as installations with more receivers.	Faster and cheaper to service, and gaps between local arrays can be more easily filled, providing much larger spatial coverage and resolution. Advantageous for studying large-scale movements of multiple species over time.

The *new array* generally showed increases in movement metrics, and new or increased knowledge of large-scale movements for several species (Sect. 3). Therefore, the compromises between single receiver locations and long-term deployments appear to be functional. The array design will likely be most beneficial for long-term deployments, increasing the capacity to capture large-scale movements and seasonal patterns over the ∼ 10-year maximum battery life of transmitters. Initial knowledge on species occurrence can provide information for further strategic deployments within the large-scale arrays. This may involve new collaborations in particular areas, or assisting in focusing available funds to service strategic stations.

“Redundancy” is an important consideration for arrays designed using the *enhanced Queensland array* model. In the current study, several receivers failed or were lost (e.g. 17 *new array* receivers). Despite some data recovery, this resulted in gaps in temporal and spatial coverage, and costs for freight and manufacturer repair. This may occur in studies more often than previously reported, as acoustic receivers that have been used for years/decades may be lost due to age or severe weather events. To protect against receiver failure or loss, sites would ideally include at least two receivers in the area (e.g., either end of a reef) or use a small node approach where three to four receivers are deployed in an area, spread out enough to increase local coverage, but close enough so that the key area is still monitored in the event of receiver failure.

For Queensland, locations were often influenced by the availability of collaborators to maintain receivers at minimal costs. Improved collaboration can generate further interest and willingness by other stakeholders in contributing to the array and further fill gaps in receiver coverage on the east coast of Australia. Results thus far suggest maintaining, if not improving (redundancy), the broad-scale Queensland array will provide sustained detections of many of the mobile species tagged with transmitters of 10-year battery life. Eastern Australia is the most rapidly changing western boundary current in the world, with species range shifts being increasingly documented [13, 14, 54]. Concerningly, there is little baseline information on population connectivity and habitat use for many marine species in this region, making predictions of resilience to anthropogenic impacts difficult. The *enhanced Queensland array* is well placed to improve our understanding of connectivity of marine species on the east coast of Australia and to contribute to the IMOS infrastructure.

The evaluation of the *enhanced Queensland array* indicates that, despite the compromises the model of maintaining receivers through a diversity of collaborators (including research institutes, government departments, tourism operators, Indigenous Ranger groups and industry) provides the infrastructure needed for long-term studies to address questions over larger spatial and longer temporal scales. The use of single receivers at locations in the *Queensland array* complements previous work evaluating the best locations for receivers to remain deployed when reducing local arrays, to maximise the contribution to broader scale networks [25, 30]. In combination, these studies can assist the design of acoustic arrays that maximise the functionality of large-scale studies in other regions. This information could also assist existing arrays increase area coverage and improvement of results aimed at describing large-scale migrations.

It is inevitable that with changing research objectives and budgets the configuration of a continental-scale array will change over time. Changing array design, in particular removing or moving receivers has usually been considered problematic when analysing data. Therefore, an ongoing challenge is dealing with changes in localised arrays while maintaining a continental-scale network [25]. For broad-scale questions such as connectivity, where movement between regions or locations can be analysed, receivers within regions/locations can be grouped into nodes [55]. A reduction or movement of receivers within nodes should subsequently have little bearing on broad-scale movement analysis. When reductions in receivers are necessary, understanding the efficiency of deployment locations can help decide which receivers to remove or move while maintaining the array’s ability to study large-scale movements [25, 30].

#### The future: next steps

Three steps are needed to maintain and maximise the value of the Queensland array. First and foremost, it is critical that a backbone of acoustic receivers remains deployed for the long-term. To address this point, IMOS has provided funding to maintain receivers at key locations for four more years, ensuring data collection can continue into the near future. Secondly, it is important to consider the issue of single receiver redundancy. This can be addressed by either deploying additional receivers in locations/regions with only few receivers, or by implementing new, smaller-scale, projects in those locations/regions. Both options are influenced by funding. To reduce the likelihood of losing data from broken receivers at single deployment locations, the newest receivers have been deployed at the most isolated sites, while at locations with multiple receivers both old and new receivers are used, until the older receivers are phased out.

The third step involves filling in the larger gaps in receiver coverage. Some of these gaps are currently being filled thanks to recent opportunities to deploy receivers at new sites. For example, DES recently deployed additional receivers at the moorings they service in the Capricorn Bunker Group (further south than the other receivers they service for the Queensland array). DES and BOF have also deployed receivers for a shark behaviour program based at one of the Capricorn Bunker Islands, North West Island (23.28°S, 151.70°E). In the northern GBR, Mike Ball Dive (tourism operator) is now maintaining additional receivers at their dive sites in the Ribbon Reefs (14.90°S, 145.68°E) for BOF and Project Manta, to target reef manta rays *Mobula alfredi*, increasing receiver coverage in the far northern section of the Queensland array. There is also a new drive to increase receiver coverage in the far northern extent of the acoustic array in northeast Queensland, including in the Torres Strait and into eastern Gulf of Carpentaria which results from collaborations between IMOS, University of Sunshine coast, industry (Rio Tinto), Indigenous Ranger groups (Mapoon), QDAF and BOF, aimed at tracking highly mobile (e.g. sailfish) and globally threatened species (sawfish, speartooth shark, hammerhead sharks, wedgefishes and giant shovelnose rays) in this biodiversity hotspot. To date, over 1000 animals have been tagged since the Queensland array project was initiated in 2020.

Importantly, the Queensland array provides infrastructure that can be used to leverage funding for new projects. For example, a project acoustically tracking sailfish movements secured funding for tags for the first-time by leveraging the Queensland array infrastructure. Other benefits from this receiver network include linking researchers tracking the same species at different locations and encouraging research groups to tag the species included in the Queensland array tagging program at their sites. Several research groups on the east coast of Australia and the Gulf of Carpentaria are currently increasing their tagging efforts on some of the species targeted by the Queensland array program. The resulting increase in number of individuals tagged over a wide spatial range will allow us to address broader-scale questions.

## Conclusion

From little things big things grow. The procurement of modest seed funds to increase receiver coverage and tagging effort in Queensland was the impetus to pull together a disparate group of stakeholders and create an extensive collaborative network that enhanced the already significant IMOS tracking infrastructure on the east coast of Australia. This included leveraging existing programs like the QDAF Shark Control Program and DES mooring maintenance to help service receivers, as well as forming new collaborations, thereby creating a program that can sustain the extended receiver coverage into the future. This collaborative momentum will lead to further benefits of future projects and additional collaborator-driven arrays.

The approach of deploying single receivers over a large spatial scale revealed previously unknown broad-scale movements, some being the largest movements recorded for a species in Australia, if not globally. This shows that the deployment of several receivers in proximity is not always required to enhance data collection (while noting that redundancy should be included in array design to ensure that large-scale movement data is not compromised if receivers are broken or lost). So far, results suggest that this state-wide installation will uncover a range of previously unknown movements that will assist in addressing key ecological, fisheries, and conservation questions for multiple species. More broadly, given the changing oceanographic process in this region [56–58], the improved spatial coverage on the east coast of Australia will provide knowledge on the effects of anthropogenic impacts and species adaptability into the Anthropocene.

### Electronic Supplementary Material

Below is the link to the electronic supplementary material.

Supplementary Material 1

## Data Availability

No datasets were generated or analysed during the current study.

## Abbreviations

IMOS ATF: Integrated Marine Observing System Animal Tracking Facility
NSW DPI: New South Wales Department of Primary Industries
AIMS: Australian Institute of Marine Science
BOF: Biopixel Oceans Foundation
CQU: Central Queensland University
DBCT: Dalrymple Bay Coal Terminal
DES: Department of Environment & Science
GPC: Gladstone Ports Corporation
JCU: James Cook University
LTMP: Long Term Monitoring Program
MBD: Mike Ball Dive Expeditions
MMP: Marine Monitoring Program
QDAF: Queensland Department of Agriculture & Fisheries
QPWS: Queensland Parks and Wildlife Service
RT: Rio Tinto
UQ: The University of Queensland
UniSC: University of the Sunshine Coast
