# Supplementary material for: From little things big things grow: enhancement of an acoustic telemetry network to monitor broad-scale movements of marine species along Australia’s east coast
Source: Mov Ecol. 2024 Apr 23;12:31. doi: 10.1186/s40462-024-00468-8 (PMC11040905; doi:10.1186/s40462-024-00468-8)
Supplement: Supplementary file 1 — Supplementary Material 1 [file 40462_2024_468_MOESM1_ESM.docx]

**Supplementary Material**

**Table S1.** Data layers used to compile broad-scale habitat, reference shapefiles and imagery for figures.

| **Data Layer Name** | **Source** | **Date** |
| --- | --- | --- |
| Australia Digital Boundary File | Australian Bureau of Statistics | 2021 |
| ESRI World Imagery Base Layer | Esri, Maxar, Earthstar Geographics and GIS User Community | 2023 |
| GBR10 GBRMP Benthic | Great Barrier Reef Marine Park Authority | 2021 |
| GBR10 GBRMP Geomorphic | Great Barrier Reef Marine Park Authority | 2021 |
| Integrated Marine and Coastal Regionalisation of Australia (IMCRA) v4.0 – Provincial Bioregions | Australian Government – Department of Climate Change, Energy, the Environment and Water | 2006 |
| Map 4 – Moreton Bay Marine Park Habitat Types and Marine National Park (green) Zones | Queensland Government Environmental Protection Agency and Department of Environment and Science | 2008 |
| Moreton Bay Broadscale Habitats | Australian Ocean Data Network 2020 | 2020 |
